# Editorial: Portraying the phytomicrobiome studies during abiotic stresses: Revisiting the past and exploring the future outcomes

**DOI:** 10.3389/fmicb.2022.1015149

**Published:** 2022-10-13

**Authors:** Kanika Khanna, Sukhmeen Kaur Kohli, Renu Bhardwaj, Anket Sharma

**Affiliations:** ^1^Department of Botanical and Environmental Sciences, Guru Nanak Dev University, Amritsar, Punjab, India; ^2^Department of Microbiology, DAV University, Jalandhar, Punjab, India; ^3^State Key Laboratory of Subtropical Silviculture, Zhejiang A&F University, Hangzhou, China

**Keywords:** phytomicrobiome, rhizosphere, signaling, symbiosis, molecular studies, phytohormones, crosstalk, omics

Plants are exposed to a plethora of climatic conditions that adversely affect their growth and development. Many different abiotic stresses can impair plants' metabolic, biochemical, and physiochemical functions, including heavy metals, salinity, flooding, freezing, excessive heat, drought, and nutrient deprivation. These conditions degrade the agro-ecosystem, ultimately ruining yields, productivity, and food supplies (Khan et al., 2021). Under these conditions, plants become disintegrated, reflecting the disrupted metabolic networks, morphological impairments, and productivity losses. Therefore, it is crucial to develop new strategies that can counteract these abiotic stresses and decode the resistance mechanisms associated with them (Kumar and Verma, 2018). This Research Topic features insights into microbiome communities during periods of stress tolerance in plants. The mechanisms unraveling plant-microbe interactions and the symbiotic associations between microbes and plants under stress are given special focus.

Plants are surrounded by different zones: namely, the phyllosphere above ground and the rhizosphere below ground. The microbial communities residing within these zones communicate with plants through an array of signaling molecules and compounds forming an effective symbiosis. Moreover, they are able to affect nutrient acquisition, and they respond well to environmental adversity (Khan et al., 2021). However, fluctuating environmental conditions can affect the resident microflora and their activities, thereby affecting plant responses. Unlocking the microbial responses toward stressful conditions will provide us with an asset for stimulating the stress resistance mechanisms in plants.

Micro-organisms provide protection to plants under harsh conditions by inducing their defense pathways and secondary metabolic profiles. They have also been used as biofertilizers, biostimulants, biopesticides, and phyto-stimulators to triggerMicro-organisms provide protection to plants under harsh conditions by inducing their defense pathways and secondary metabolic profiles. They have also been used as biofertilizers, biostimulants, biopesticides, and phyto-stimulators to trigger the systemic resistance mechanisms in plants, along with modulating their hormonal profiles and signaling crosstalk among plant hormones. Microbe-mediated stress resistance is chaperoned to plants *via* crucial crosstalk among diverse molecular, cellular, and physiological regulatory networks (Enebe and Babalola, 2018) (see Figure 1).

**Figure 1:**
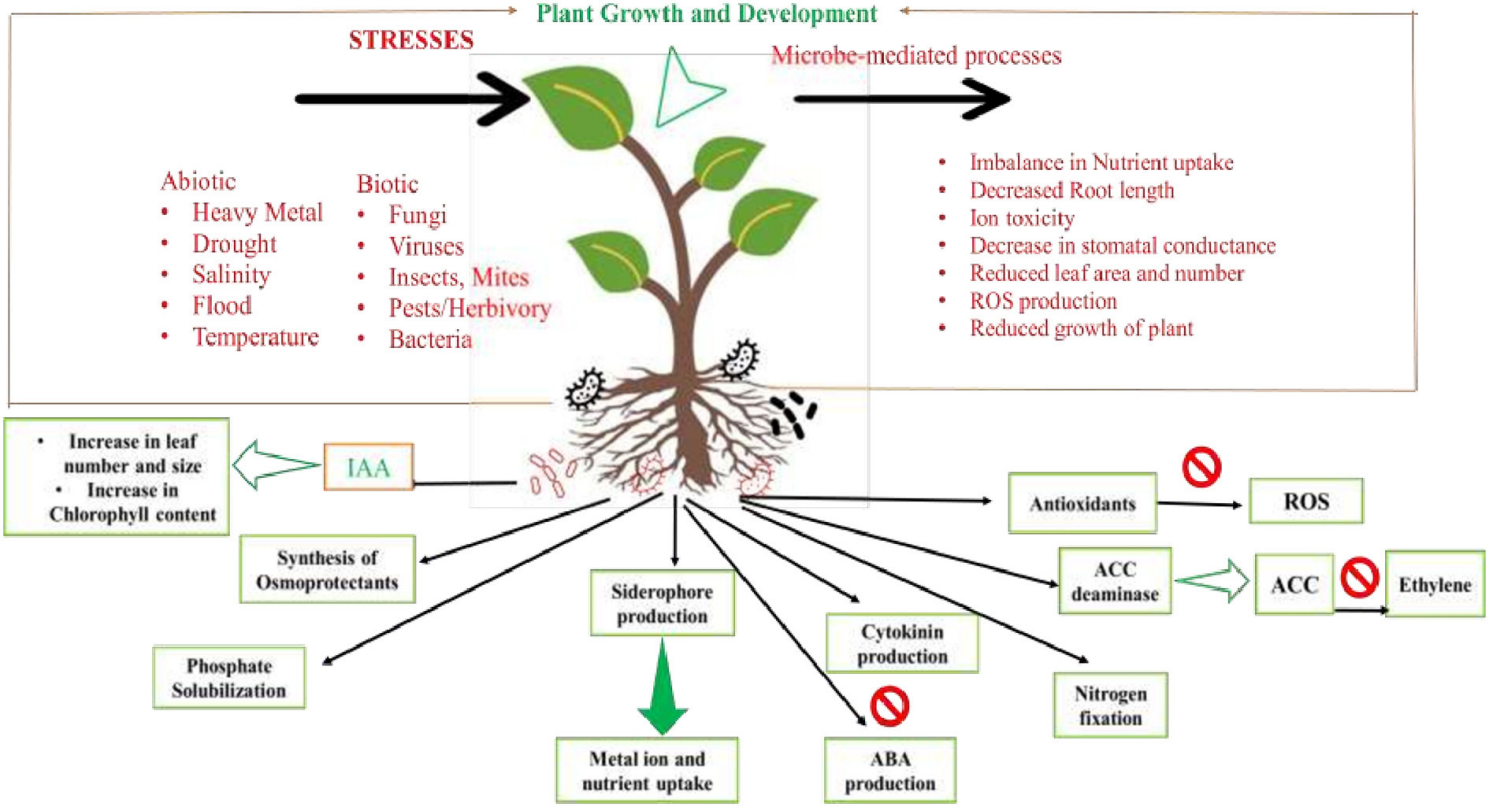
Schematic representation of the microbial dynamics within soil during stresses. Microbes play an essential role in plant growth and development during adverse conditions through inducing defense signaling and stress-responsive processes in plants.

Microbial communities in close proximity to plants are called phytomicrobiomes. In this collective microflora, various microbial forms, such as bacteria, fungi, actinobacteria, and archaebacteria, co-exist and form a complete holobiont. The exact makeup of the plant microbiome is greatly influenced by the behavior of the microbes, as well as their genotype and life cycle, the architecture and physicochemical properties of the soil, climatic factors, and various other edaphic factors, along with the presence of abiotic as well as biotic stressors (Liu et al., 2020). Common abiotic factors that affect the microbial niche include the presence of pesticides and heavy metals, temperature, and water-logging conditions. A unique kind of communication takes place within the rhizosphere, rhizoplane, and plant roots that tends to mediate several morphological, genotypic, and biochemical modulations within plants in order to mitigate stresses *via* different mechanisms (Majeed et al., 2018).

It has been found that microbes release siderophores and phytohormones; enable phosphate solubilization, nitrogen fixation, and nutrient acquisition; and show ACC-deaminase activity and antioxidant defense responses during stresses. Awan et al. surveyed the role of the biocontrol agent *Bacillus subtilis* in combination with vital micronutrients, nitrogen, phosphorous, potassium, and zinc against early blight disease caused by *Alternaria solani* in tomato plants. Their quantitative PCR analysis revealed that the interaction between *B. subtilis* and plant nutrients improved crop resilience in infected plants by strengthening the antioxidant defense responses, along with inducing the nutritive levels of the crop and boosting the chlorophyll, carotenoid, and total phenolic content. Moreover, the pathogen load was also lowered, demonstrating the synergies between both actors and confirming *B. subtilis* as a most suitable alternative to chemical fungicide for sustainable agriculture.

Another important mechanism for the defense process is the release of the numerous lytic enzymes, volatile organic compounds, phenolic compounds, and metabolites that act as signaling compounds during the defense signaling cascade against both pathogens and abiotic stressors (Shah et al., 2021). They also target the acquired systemic resistance mechanisms in plants and induce them to employ specific defensive strategies to overcome the adverse condition. Mulk et al. *Bacillus* spp., a highly significant bacterial species with antagonistic activity against four root pathogens targeting wheat plants: *Fusarium oxysporum, F. moniliforme, Rhizoctonia solani*, and *Macrophomina phaseolina*. Wheat plants inoculated with *Bacillus* strains also showed lowered electrolyte leakage and increased relative water content in comparison to control plants, thus supporting the potential of *Bacillus* spp. as a biocontrol agent.

The most significant classical example of phytomicrobiome signaling in plants is the association of the rhizobia-legume symbiotic association and mycorrhizal interaction with the roots of plants (Shah et al., 2021). Moreover, many plant-growth-promoting bacteria (PGPR) also reside in free form within the rhizosphere-plant proximity. They also enhance the microbial niche with their secretions, which activate chemical signaling cascades for plant growth and stress mitigation. The findings of other types of -omics investigations (i.e., metabolomics, proteomics, transcriptomics, secretomics, phenomics, interactomics, glycomics), along with examinations of phytohormonal signaling processes and hormonal crosstalk, form a roadmap for phytomicrobiome studies (Liu et al., 2020). These studies have paved new paths for exploring the investigations and discoveries within plant microbiomes and their external and internal communications.

The role of microbial populations as biocontrol agents against different pathogenic organisms and as biofertilizers has been well-depicted (Trivedi, 2021). Shoaib et al. investigated the roles of zinc and green manure in controlling the incidence of *Macrophomina phaseolina* infection, which causes charcoal rot disease in mug bean. Both of these substances induced plant resistance and improved growth, photosynthesis, total proteins, physiology, biomass, harvest index, and yield, while reducing disease incidence. Moreover, the production of antioxidants was also triggered, as seen in the levels of polyphenol oxidase, superoxide dismutase, catalase, and peroxidase. Ultimately, these interventions alleviated disease and increased profits by improving the harvest index and cost-benefit ratio.

Therefore, we have focused on the importance of microbes for sustainable agriculture and their applicability in the field through commercialization. Additionally, different gene and cellular responses, including transcript levels, proteins, metabolic profiles, and signaling pathways in this network, are also modulated in phytomicrobiomes experiencing stress. The series of primary and secondary effects, as explored in these studies of the phytomicrobiome under harsh conditions, are a most crucial step for further investigation.

## Author contributions

KK drafted the editorial text. SK and AS revised the draft. KK and RB finalized the editorial text and approved the final version. All authors contributed to the article and approved the submitted version.

## Conflict of interest

The authors declare that the research was conducted in the absence of any commercial or financial relationships that could be construed as a potential conflict of interest.

## Publisher's note

All claims expressed in this article are solely those of the authors and do not necessarily represent those of their affiliated organizations, or those of the publisher, the editors and the reviewers. Any product that may be evaluated in this article, or claim that may be made by its manufacturer, is not guaranteed or endorsed by the publisher.
